# Postpandemic Change in Demographic and Clinical Features of Patients With Omicron Who Were Hospitalized: Territory-Wide Retrospective Repeated Cross-Sectional Study in Hong Kong

**DOI:** 10.2196/75635

**Published:** 2026-02-09

**Authors:** Christie J Y Ching, Sunny C L Chan, Teddy T L Lee, Hugo H H Pui, Bosco K H Leung, Man Sing Wong, Tafu Yamamoto, Chak Kwan Tong, Cantian Wang, Timothy H Rainer, Abraham K C Wai

**Affiliations:** 1 Department of Emergency Medicine, School of Clinical Medicine, University of Hong Kong, G06, G/F, University of Hong Kong the Hong Kong Jockey Club Building for Interdisciplinary Research, 5 Sassoon Road, Pokfulam, Hong Kong, China, 85239179175; 2 Department of Land Surveying and Geo-informatics, Faculty of Construction and Environment, The Hong Kong Polytechnic University, Hong Kong, China; 3 Department of Accident & Emergency, Yan Chai Hospital, Hong Kong, China; 4 Intensive Care Unit, Tuen Mun Hospital, Hong Kong, China; 5 Department of Accident & Emergency, University of Hong Kong - Shenzhen Hospital, Shenzhen, China; 6 Department of Accident & Emergency, Queen Mary Hospital, Hong Kong, China

**Keywords:** emergency department, COVID-19, infectious disease epidemiology, Omicron, SARS-CoV-2

## Abstract

**Background:**

The Omicron variant of SARS-CoV-2 underwent several mutations since it was first identified in November 2021, with a large outbreak in Hong Kong in early 2022. Yet, local cases of Omicron infections persist, even though the COVID-19 pandemic ended in May 2023.

**Objective:**

This study aims to describe the changes in demographic and clinical characteristics of patients infected with COVID-19 across different Omicron waves in Hong Kong and determine whether the changes continued into the postpandemic period.

**Methods:**

This retrospective repeated cross-sectional study collected data on patients infected with COVID-19 admitted to public hospitals in Hong Kong between May 1, 2022, and May 31, 2024. These data were later categorized into 3 periods based on the Omicron strain. A subsequent age-stratified descriptive analysis was conducted on each characteristic to identify any significant differences across the periods.

**Results:**

First, the case fatality ratio significantly lowered among those older than 85 years (1.5% proportion decrease, period 1: 11.6%, period 2: 10.1%, effect size: 0.02; *P*<.001). Second, most patients were Chinese (≥68.7% per age group and period), and females were predominantly infected for those aged older than 85 years (≥56.9% per period). Third, the Charlson Comorbidity Index scores in most age groups showed a predominant proportion of infected individuals with 0 scores (more than 70% per period). Fourth, most cases were from slightly disadvantaged populations in Hong Kong (≥30.5% per age group per period). Fifth, clinical management of Omicron hospitalizations showed lowered length of hospital stays among adults and older individuals (≥1 d decrease between periods 1 and 3, per age group), as well as increased administration of bronchodilators.

**Conclusions:**

Despite the decreasing incidence of Omicron cases admitted to public hospitals in Hong Kong, the increasing case fatality ratio with age suggests that long-term surveillance of COVID-19 should be maintained to prepare for potential mutations and outbreaks.

## Introduction

The Omicron outbreak in early 2022 resulted in a crude population mortality rate of 37.7 per million, overwhelming the Hong Kong health system [1,2]. On May 5, 2023, the World Health Organization (WHO) officially declared an end to the pandemic [3]. Nevertheless, reports of COVID-19 outbreaks persisted in Hong Kong and globally [4,5]. By May 2024, the Communicable Disease Branch of the Center for Health Protection in Hong Kong reported that JN.1, the local dominant SARS-CoV-2 variant, did not cause more severe disease compared to XBB and its descendant lineages, the previous dominant strain [6].

Postpandemic surveillance is crucial for understanding the ongoing impact of COVID-19 and improving future pandemic preparedness. Current literature on the post–COVID-19 era primarily focuses on COVID-19 (long COVID-19) and delayed health care for patients with noncommunicable diseases during the pandemic. Long COVID-19 is a multisystemic condition manifesting as new onset cardiovascular disease, thrombotic disease, cerebrovascular disease, myalgic encephalomyelitis or chronic fatigue syndrome, type 2 diabetes, or postural orthostatic tachycardia syndrome [7]. Delayed health care for patients with noncommunicable diseases has resulted in complications and multimorbidity, contributing to increased health care costs [8,9].

An updated characterization of hospitalized patients with COVID-19 can provide a clearer clinical picture after the COVID-19 pandemic. Previous studies have identified several factors associated with worse prognosis in hospitalized patients with COVID-19, including blood biomarkers [10-13], hypertensive and diabetic medications [14,15], older age [16,17], males [12,13,17], hypertension and diabetes comorbidities [18,19], frailty [20,21], length of hospital stay [22], and lower socioeconomic background [23,24]. A longitudinal investigation into these parameters may enhance our understanding of the ongoing impact of COVID-19.

The aim of this study is to describe the changes in demographic and clinical characteristics of hospitalized patients infected with Omicron across different waves in Hong Kong, and to determine whether these changes continued into the postpandemic period. By conducting these comparisons, we provide insights into the evolving clinical profile of COVID-19 to inform the development of future public health strategies.

## Methods

### Participants and Context

This study used a territory-wide, retrospective, repeated, cross-sectional study design to collect electronic clinical data from May 1, 2022, to May 31, 2024, sourced from the Clinical Data Analysis and Reporting System, managed by the Hong Kong Hospital Authority [25]. The Clinical Data Analysis and Reporting System encompasses data from 18 public hospitals in Hong Kong and has been validated in previous studies for its reliability [26]. Also, the patients included in this study were identified through confirmatory laboratory test results (reverse transcription polymerase chain reaction, multiplex polymerase chain reaction, and polymerase chain reaction) conducted in hospitals or government public health laboratories during the study period.

**Figure 1.**
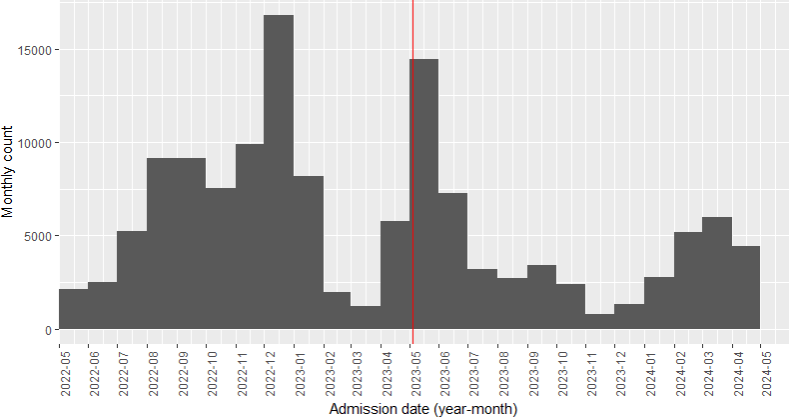
A histogram plot showing the monthly episodes of patients infected with Omicron hospitalized in Hong Kong between 2022 and 2024 in this repeated cross-sectional study (total episodes=136,544). The red line indicates the end of the pandemic.

The study period was divided into 3 groups according to the epidemic waves illustrated in Figure 1:

1. Period 1: between May 1, 2022, and February 28, 2023.
2. Period 2: between March 1, 2023, and November 30, 2023.
3. Period 3: between December 1, 2023, and May 31, 2024.

Based on WHO’s declaration of an end to the pandemic on May 5, 2023, [3], changes that continued into the postpandemic period were identified by differences in characteristics between periods 2 and 3. In addition, this study adopted episode count as the primary unit of measurement. An episode was defined as a single hospital admission to discharge occurrence, regardless of readmission of unique patients.

### Defining Demographic Characteristics

This study categorized ages into 5 groups: 0‐17, 18‐64, 65‐74, 75‐84, and ≥85 years. Socioeconomic Deprivation Index (SDI) was defined using the deprivation score in each tertiary planning unit in Hong Kong [27]. The score was calculated based on the 2021 population census, considering factors such as marital status, school attendance, working population, monthly domestic household income, household size, and tenure of accommodation [28]. Charlson Comorbidity Index (CCI) score was calculated using *International Statistical Classification of Diseases and Related Health Problems, 10th Revision* (*ICD-10*) codes [29], and categorized into 4 groups: 0, 1, 2‐3, and ≥4. Specific comorbidities were identified, including unspecified essential hypertension (*ICD-10*: I10), type 2 diabetes mellitus (*ICD-10*: E11.0-E11.9), and unspecified hyperlipidemia (*ICD-10*: E78.5). Frailty-related episodes were identified based on co-occurring *ICD-10* diagnoses related to frailty markers, validated in a previous study [30]. The case fatality ratio (CFR) was defined, according to the WHO definition, as the proportion of COVID-19–related deaths per confirmed case of COVID-19 [31].

### Defining Clinical Characteristics

All in-patient drugs administered during each episode were categorized according to Table S2 in Multimedia Appendix 1. For the biomarkers of interest, the earliest laboratory requests made during each episode were obtained to reflect the biomarker value closest to the date of COVID-19 diagnosis. For biomarkers with less than 25% missing data, multiple imputation using k-nearest neighbors with Gower Distance was applied [32]. Also, patients with more than 2 hospital admissions were used to measure readmission.

### Descriptive Analysis

Age-stratified descriptive analyses were used to characterize the changes in demographic and clinical characteristics across distinct pandemic periods. Categorical variables (≥2 hospital readmissions, sex, comorbidities, race, CCI score, frailty-related episodes, Social Deprivation Index, and drug administration) were presented as frequency (n, %). Continuous variables (length of stay and blood biomarkers) were reported as median (IQR).

To account for varying period durations, the measurements were standardized: categorical variables as episodes per month and continuous variables as mean values per month.

Continuous variables across periods were compared using the Kruskal-Wallis test; categorical variables were compared using the proportions test (Fisher exact test was used if the proportions test assumptions were violated). Post hoc pairwise comparisons of significant differences were performed (proportions test or Fisher exact test for categorical, Dunn test for continuous variables), with Bonferroni correction for multiple testing.

The effect sizes for significant variables were measured using Cramér V for categorical and Eta squared test for continuous variables. All *P* values were 2-sided; statistical significance was defined as *P*<.05. Analyses were conducted with R (version 4.2.2; R Core Team) [33].

### Ethical Considerations

This study received ethical approval from The University of Hong Kong Institutional Review Board (UW 20‐112). Informed consent was waived by the institutional review board as all the patients’ data were collected anonymously. The privacy and confidentiality of human subjects were protected by maintaining anonymity, not collecting personal data, and ensuring that data were securely stored by the principal investigator and deleted after the storage period. This study was conducted in accordance with the Declaration of Helsinki, and no compensation was provided for the human subjects involved.

## Results

### Hospitalization and Epidemic Trends

Between May 2022 and May 2024, Figure 1 and Table 1 illustrated the 3 distinct epidemic curves corresponding to periods 1-3, each exhibiting progressively lower peaks. This trend was further highlighted in Tables 2-6, which showed depleting total number of hospitalized Omicron cases across the study period among all age groups: 0‐17 years (75.2% decrease, Period 1: 8399, Period 3: 2081); 18‐64 years (71.6% decrease, Period 1: 15,274, Period 3: 4340); 65‐74 years (69.9% decrease, Period 1: 13,549, Period 3: 4072); 75‐84 years (67.5% decrease, Period 1: 15,622, Period 3: 5076); and older than 85 years (62.8% decrease, Period 1: 19,717, Period 3: 7337). The difference between periods 1 and 3 diminished as the age group got older. Moreover, the rate of hospital readmissions per month decreased across all ages: 0‐17 years (14.8 episodes per month decrease, Period 1: 41.6 episodes per month, Period 3: 26.8 episodes per month); 18‐64 years (225.8 episodes per month decrease, Period 1: 296.0 episodes per month, Period 3: 70.2 episodes per month); 65‐74 years (249.8 episodes per month decrease, Period 1: 346.8 episodes per month, Period 3: 97 episodes per month); 75‐84 years (332 episodes per month decrease, Period 1: 484.5 episodes per month, Period 3: 152.5 episodes per month); and older than 85 years (480.8 episodes per month decrease, Period 1: 754 episodes per month, Period 3: 273.2 episodes per month).

**Table 1.** Frequency of hospitalized patients infected with Omicron in Hong Kong from this repeated cross-sectional study (2022-2024) (total episodes, N=136,544).

Date	Values, n (%)
May 1, 2022	2076 (1.5)
June 1, 2022	2447 (1.8)
July 1, 2022	5202 (3.8)
January 8, 2022	8929 (6.5)
September 1, 2022	9314 (6.8)
October 1, 2022	7483 (5.5)
November 1, 2022	9735 (7.1)
December 1, 2022	16,695 (12.2)
January 1, 2023	8492 (6.2)
February 1, 2023	2098 (1.5)
March 1, 2023	1165 (0.9)
April 1, 2023	5487 (4)
May 1, 2023	14,395 (10.5)
June 1, 2023	7487 (5.5)
July 1, 2023	3276 (2.4)
August 1, 2023	2654 (1.9)
January 9, 2023	3379 (2.5)
October 1, 2023	2484 (1.8)
November 1, 2023	840 (0.6)
December 1, 2023	1246 (0.9)
January 1, 2024	2757 (2)
February 1, 2024	5039 (3.7)
March 1, 2024	6077 (4.5)
April 1, 2024	4407 (3.2)
May 1, 2024	3380 (2.5)

**Table 2.** Characteristics of patients infected with Omicron in Hong Kong from this repeated cross-sectional study (2022‐2024), stratified by ages 0‐17 years (total episodes, N=14,556).

Characteristics	Period 1^a^		Period 2^b^		Period 3^c^		*P* value	Effect size
	Observed	Adjusted	Observed	Adjusted	Observed	Adjusted		
Case fatality ratio	0.1	—^d^	0.1	—	0	—	—	—
≥2 hospital readmissions, n (%)	416 (5)	41.6 (0.5)	250 (6.1)	27.8 (0.68)	161 (7.7)	26.8 (1.28)	<.001^e^	0.89^f^
Sex, n (%)								
Male	4689 (55.8)	468.9 (5.58)	2167 (53.2)	240.8 (5.91)	1128 (54.2)	188 (9.03)	.02^e^	0.02^f^
Female	3710 (44.2)	371 (4.42)	1909 (46.8)	212.1 (5.2)	953 (45.8)	158.8 (7.63)	.02^e^	0.02^f^
Comorbidities, n (%)								
Essential primary hypertension	1 (0)	0.1 (0)	0 (0)	0 (0)	0 (0)	0 (0)	.37^g^	—
Type 2 diabetes mellitus	3 (0)	0.3 (0)	2 (0)	0.2 (0)	0 (0)	0 (0)	.69^g^	—
Hyperlipidemia (unspecified)	1 (0)	0.1 (0)	0 (0)	0 (0)	0 (0)	0 (0)	>.99^g^	—
Race, n (%)								
Chinese	6483 (77.2)	648.3 (7.72)	3035 (74.5)	337.2 (8.28)	1429 (68.7)	238.2 (11.45)	<.001^e^	0.07^f^
Non-Chinese	1916 (22.8)	191.6 (2.28)	1041 (25.5)	115.7 (2.83)	652 (31.3)	108.7 (5.21)	<.001^e^	0.07^f^
Length of hospital stay (days), median (IQR)	2 (2)	3.19 (0.77)	2 (2)	2.79 (0.83)	2 (2)	2.56 (0.54)	<.001^h^	0.009^i^
Charlson Comorbidity Index score, n (%)								
0	8236 (98.1)	823.6 (9.81)	3962 (97.2)	440.2 (10.8)	2023 (97.6)	337.2 (16.26)	.003^e^	0.03^f^
1	69 (0.8)	6.9 (0.08)	47 (1.2)	5.2 (0.13)	18 (0.9)	3 (0.15)	.18^e^	—
2‐3	87 (1)	8.7 (0.1)	63 (1.5)	7 (0.17)	28 (1.4)	4.7 (0.23)	.04^e^	0.02^f^
≥4	5 (0.1)	0.5 (0.01)	4 (0.1)	0.4 (0.01)	3 (0.1)	0.5 (0.02)	.36^g^	—
Frailty-related episodes, n (%)	58 (0.7)	5.8 (0.07)	24 (0.6)	2.7 (0.07)	8 (0.4)	1.3 (0.07)	.26^e^	—
Social deprivation index, n (%)								
1 (least disadvantaged)	1146 (14)	114.6 (1.4)	620 (15.3)	68.9 (1.7)	309 (14.9)	51.5 (2.48)	.04^e^	0.02^f^
2 (slightly disadvantaged)	2935 (35.7)	293.5 (3.57)	1477 (36.4)	164.1 (4.04)	750 (36.1)	125 (6.02)	.31^e^	—
3 (moderately disadvantaged)	2439 (29.7)	243.9 (2.97)	1070 (26.4)	118.9 (2.93)	544 (26.2)	90.7 (4.37)	<.001^e^	0.03^f^
4 (most disadvantaged)	1695 (20.6)	169.5 (2.06)	891 (22)	99 (2.44)	474 (22.8)	79 (3.8)	.01^e^	0.02^f^
Drug administration, n (%)								
Angiotensin-converting enzyme inhibitors	23 (0.3)	2.3 (0.03)	12 (0.3)	1.3 (0.03)	7 (0.3)	1.2 (0.05)	.89^e^	—
Antidiabetics	29 (0.3)	2.9 (0.03)	16 (0.4)	1.8 (0.04)	8 (0.4)	1.3 (0.07)	.91^e^	—
Antiplatelets and anticoagulants	60 (0.7)	6 (0.07)	48 (1.2)	5.3 (0.13)	25 (1.2)	4.2 (0.2)	.01^e^	0.02^f^
Beta blockers	26 (0.3)	2.6 (0.03)	5 (0.1)	0.6 (0.01)	1 (0)	0.2 (0.0)	.02^g^	0.02^f^
Bronchodilators	243 (2.9)	24.3 (0.29)	220 (5.4)	24.4 (0.6)	148 (7.1)	24.7 (1.18)	<.001^e^	0.08^f^
Calcium channel blocker	14 (0.2)	1.4 (0.02)	17 (0.4)	1.9 (0.04)	2 (0.1)	0.3 (0.02)	.01^g^	0.02^f^
Diuretics	49 (0.6)	4.9 (0.06)	42 (1)	4.7 (0.11)	20 (1)	3.3 (0.17)	.01^e^	0.02^f^
Inhaled corticosteroids	174 (2.1)	17.4 (0.21)	114 (2.8)	12.7 (0.31)	87 (4.2)	14.5 (0.7)	<.001^e^	0.04^f^
Rheumatoid drugs	21 (0.3)	2.1 (0.03)	17 (0.4)	1.9 (0.04)	5 (0.2)	0.8 (0.03)	.24^e^	—
Statins	1 (0)	0.1 (0)	3 (0.1)	0.3 (0.01)	0 (0)	0 (0)	.17^g^	—
Systemic corticosteroids	715 (8.5)	71.5 (0.85)	400 (9.8)	44.4 (1.09)	189 (9.1)	31.5 (1.52)	.06^e^	—
Blood biomarkers, median (IQR)								
Albumin (g/L)	38.82 (3.98)	40.4 (0.3)	38.82 (3.18)	39.8 (0.1)	38.82 (3.58)	39.9 (0.2)	<.001^h^	0.003^i^
Neutrophil (×10^9^/L)	4.48 (1.27)	4.4 (0.2)	4.48 (1.46)	4.6 (0.1)	4.48 (1.75)	4.8 (0.1)	<.001^h^	0.0009^i^
Bilirubin (µmol/L)	9.30 (14.20)	12.4 (0.6)	9.03 (14.10)	13 (0.6)	8.80 (14.10)	12.6 (0.3)	.67^h^	—
Lymphocyte (×10^9^/L)	0.86 (0.89)	1.6 (0.2)	1 (1.44)	1.9 (0.2)	1.40 (2.14)	2.3 (0.04)	<.001^h^	0.02^i^
Platelet (×10^9^/L)	189 (127.50)	216 (7.5)	211 (155.25)	234.6 (7.5)	232 (182)	252.2 (3.2)	<.001^h^	0.01^i^

a Episodes=8399 and head count=8179.

b Episodes=4076 and head count=3940.

c Episodes=2081 and head count=1995.

d Not available.

e Proportions test.

f Cramér V test.

g Fisher exact test.

h Kruskal-Wallis test.

i Eta squared test.

**Table 3.** Characteristics of patients infected with Omicron in Hong Kong from this repeated cross-sectional study (2022‐2024), stratified by ages 18‐64 years (total episodes, N=28,183).

Characteristics	Period 1^a^		Period 2^b^		Period 3^c^		*P* value	Effect size
	Observed	Adjusted	Observed	Adjusted	Observed	Adjusted		
Case fatality ratio	2.7	—^d^	2.9	—	2.6	—	—	—
≥2 hospital readmissions, n (%)	2960 (19.4)	296 (1.94)	936 (10.9)	104 (1.21)	421 (9.7)	70.2 (1.62)	<.001^e^	0.72^f^
Sex, n (%)								
Male	7523 (49.3)	752.3 (4.93)	3984 (46.5)	442.7 (5.17)	2053 (47.3)	342.2 (7.88)	<.001^e^	0.02^f^
Female	7751 (50.7)	775.1 (5.07)	4585 (53.5)	509.4 (5.94)	2287 (52.7)	381.2 (8.78)	<.001^e^	0.02^f^
Comorbidities, n (%)								
Essential primary hypertension	432 (2.8)	43.2 (0.28)	286 (3.3)	31.8 (0.37)	161 (3.7)	26.8 (0.62)	.005^e^	0.02^f^
Type 2 diabetes mellitus	465 (3)	46.5 (0.3)	290 (3.4)	32.2 (0.38)	122 (2.8)	20.3 (0.47)	.16^e^	—
Hyperlipidemia (unspecified)	173 (1.1)	17.3 (0.11)	110 (1.3)	12.2 (0.14)	47 (1.1)	7.8 (0.18)	.49^e^	—
Race, n (%)								
Chinese	13,868 (90.8)	1386.8 (9.08)	7801 (91)	866.8 (10.1)	3928 (90.5)	654.7 (15.08)	.60^e^	—
Non-Chinese	1406 (9.2)	140.6 (0.92)	768 (9)	85.3 (1)	412 (9.5)	68.7 (1.58)	.60^e^	—
Length of hospital stay (days), median (IQR)	4 (6)	11 (2.7)	3 (5)	10 (3)	3 (4)	6.9 (2.3)	<.001^g^	0.007^h^
Charlson Comorbidity Index score, n (%)								
0	11,950 (78.5)	1195 (7.85)	6657 (78)	739.7 (8.67)	3248 (76.5)	541.3 (12.75)	<.001^e^	0.03^f^
1	1160 (7.6)	116 (0.76)	744 (8.7)	82.7 (0.97)	405 (9.5)	67.5 (1.58)	<.001^e^	0.02^f^
2‐3	1644 (10.8)	164.4 (1.08)	848 (9.9)	94.2 (1.1)	444 (10.5)	74 (1.75)	.10^e^	—
≥4	471 (3.1)	47.1 (0.31)	287 (3.4)	31.9 (0.38)	150 (3.5)	25 (0.58)	.34^e^	—
Frailty-related episodes, n (%)	443 (2.9)	44.3 (0.29)	272 (3.2)	30.2 (0.36)	124 (2.9)	20.7 (0.48)	.43^e^	—
Social deprivation index, n (%)								
1 (least disadvantaged)	2148 (14.3)	214.8 (1.43)	1118 (13.1)	124.2 (1.46)	573 (13.3)	95.5 (2.22)	.06^e^	—
2 (slightly disadvantaged)	5241 (34.9)	524.1 (3.49)	3042 (35.7)	338 (3.97)	1541 (35.7)	256.8 (5.95)	.11^e^	—
3 (moderately disadvantaged)	4343 (28.9)	434.3 (2.89)	2449 (28.7)	272.1 (3.19)	1222 (28.3)	203.7 (4.72)	.88^e^	—
4 (most disadvantaged)	3283 (21.9)	328.3 (2.19)	1915 (22.5)	212.8 (2.5)	977 (22.7)	162.8 (3.78)	.18^e^	—
Drug administration, n (%)								
Angiotensin-converting enzyme inhibitors	2496 (16.3)	249.6 (1.63)	1559 (18.2)	173.2 (2.02)	802 (18.5)	133.7 (3.08)	<.001^e^	0.02^f^
Antidiabetics	2627 (17.2)	262.7 (1.72)	1682 (19.6)	186.9 (2.18)	822 (18.9)	137 (3.15)	<.001^e^	0.03^f^
Antiplatelets and anticoagulants	4311 (28.2)	431.1 (2.82)	2526 (29.5)	280.7 (3.28)	1275 (29.4)	212.5 (4.9)	.08^e^	—
Beta blockers	2046 (13.4)	204.6 (1.34)	332 (3.9)	36.9 (0.43)	40 (0.9)	6.7 (0.15)	<.001^e^	0.52^f^
Bronchodilators	1017 (6.7)	101.7 (0.67)	779 (9.1)	86.6 (1.01)	530 (12.2)	88.3 (2.03)	<.001^e^	0.07^f^
Calcium channel blocker	3406 (22.3)	340.6 (2.23)	2060 (24)	228.9 (2.67)	1079 (24.9)	179.8 (4.15)	<.001^e^	0.02^f^
Diuretics	1760 (11.5)	176 (1.15)	1074 (12.5)	119.3 (1.39)	613 (14.1)	102.2 (2.35)	<.001^e^	0.03^f^
Inhaled corticosteroids	783 (5.1)	78.3 (0.51)	479 (5.6)	53.2 (0.62)	279 (6.4)	46.5 (1.07)	<.001^e^	0.02^f^
Rheumatoid drugs	881 (5.8)	88.1 (0.58)	704 (8.2)	78.2 (0.91)	336 (7.7)	56 (1.28)	<.001^e^	0.04^f^
Statins	3038 (19.9)	303.8 (1.99)	2026 (23.6)	225.1 (2.62)	1039 (23.9)	173.2 (3.98)	<.001^e^	0.05^f^
Systemic corticosteroids	2867 (18.8)	286.7 (1.88)	2330 (27.2)	258.9 (3.02)	1113 (25.6)	185.5 (4.27)	<.001^e^	0.09^f^
Blood biomarkers, median (IQR)								
Albumin (g/L)	38.70 (7.10)	37.3 (0.9)	38 (7.50)	36.9 (0.3)	37.90 (8)	36.7 (0.4)	<.001^g^	0.002^h^
Neutrophil (×10^9^/L)	4.48 (3.13)	5.6 (0.4)	4.90 (3.58)	5.8 (0.1)	5.12 (3.91)	6.4 (0.2)	<.001^g^	0.005^h^
Bilirubin (µmol/L)	8 (7.50)	11.6 (0.7)	7.90 (6.60)	10.8 (0.5)	8 (6.70)	11 (0.7)	<.001^g^	0.001^h^
Lymphocyte (×10^9^/L)	1 (0.89)	1.5 (0.06)	0.96 (0.87)	1.6 (0.1)	1.04 (0.96)	1.7 (0.2)	<.001^g^	0.002^h^
Platelet (×10^9^/L)	208 (109)	221.5 (6)	206 (104)	220.5 (4.6)	217 (115)	227.8 (4.7)	<.001^g^	0.001^h^

a Episodes=15,274 and head count=13,626.

b Episodes=8569 and head count=8063.

c Episodes=4340 and head count=4107.

d Not available.

e Proportions test.

f Cramér V test.

g Kruskal-Wallis test.

h Eta squared test.

**Table 4.** Characteristics of patients infected with Omicron in Hong Kong from this repeated cross-sectional study (2022‐2024), stratified by ages 65‐74 years (total episodes, N=25,045).

Characteristics	Period 1^a^		Period 2^b^		Period 3^c^		*P* value	Effect size
	Observed	Adjusted	Observed	Adjusted	Observed	Adjusted		
Case fatality ratio	4.9	—^d^	5.2	—	5.5	—	—	—
≥2 hospital readmissions, n (%)	3468 (25.8)	346.8 (2.58)	1074 (14.5)	119.3 (1.61)	582 (14.3)	97 (2.38)	<.001^e^	0.64^f^
Sex, n (%)								
Male	8190 (60.9)	819 (6.09)	4287 (57.7)	476.3 (6.41)	2445 (60)	407.5 (10)	<.001^e^	0.02^f^
Female	5269 (39.1)	526.9 (3.91)	3137 (42.3)	348.6 (4.7)	1627 (40)	271.2 (6.67)	<.001^e^	0.02^f^
Comorbidities, n (%)								
Essential primary hypertension	662 (4.9)	66.2 (0.49)	445 (6)	49.4 (0.67)	227 (5.6)	37.8 (0.93)	.002^e^	0.02^f^
Type 2 diabetes mellitus	677 (5)	67.7 (0.5)	466 (6.3)	51.8 (0.7)	191 (4.7)	31.8 (0.78)	<.001^e^	0.08^f^
Hyperlipidemia (unspecified)	294 (2.2)	29.4 (0.22)	215 (2.9)	23.9 (0.32)	106 (2.6)	17.7 (0.43)	.004^e^	0.02^f^
Race, n (%)								
Chinese	12,876 (95.7)	1287.6 (9.57)	7124 (96)	791.6 (10.67)	3894 (95.6)	649 (15.93)	.006^e^	0.02^f^
Non-Chinese	583 (4.3)	58.3 (0.43)	300 (4)	33.3 (0.44)	178 (4.4)	29.7 (0.73)	.006^e^	0.02^f^
Length of hospital stay (days), median (IQR)	6 (7)	10.4 (1.3)	4 (7)	9.1 (1.8)	4 (6)	7.8 (2.3)	<.001^g^	0.01^h^
Charlson Comorbidity Index score, n (%)								
0	9740 (72.5)	974 (7.25)	5286 (71.3)	587.3 (7.92)	2845 (71.2)	474.2 (11.87)	.04^e^	0.01^f^
1	1706 (12.7)	170.6 (1.27)	1072 (14.5)	119.1 (1.61)	551 (13.8)	91.8 (2.3)	<.001^e^	0.02^f^
2‐3	1516 (11.3)	151.6 (1.13)	769 (10.4)	85.4 (1.16)	432 (10.8)	72 (1.8)	.16^e^	—
≥4	479 (3.6)	47.9 (0.36)	282 (3.8)	31.3 (0.42)	169 (4.2)	28.2 (0.7)	.17^e^	—
Frailty-related episodes, n (%)	674 (5)	67.4 (0.5)	490 (6.6)	54.4 (0.73)	218 (5.4)	36.3 (0.9)	<.001^e^	0.03^f^
Social deprivation index, n (%)								
1 (least disadvantaged)	1788 (13.3)	178.8 (1.33)	996 (13.5)	110.7 (1.5)	544 (13.4)	90.7 (2.23)	.90^e^	—
2 (slightly disadvantaged)	4879 (36.4)	487.9 (3.64)	2636 (35.6)	292.9 (3.96)	1465 (36.1)	244.2 (6.02)	.76^e^	—
3 (moderately disadvantaged)	3901 (29.1)	390.1 (2.91)	2119 (28.6)	235.4 (3.18)	1184 (29.2)	197.3 (4.87)	.83^e^	—
4 (most disadvantaged)	2826 (21.1)	282.6 (2.11)	1649 (22.3)	183.2 (2.48)	868 (21.4)	144.7 (3.57)	.07^e^	—
Drug administration, n (%)								
Angiotensin-converting enzyme inhibitors	4496 (33.4)	449.6 (3.34)	2545 (34.3)	282.8 (3.81)	1342 (33)	223.7 (5.5)	.20^e^	—
Antidiabetics	4454 (33.1)	445.4 (3.31)	2631 (35.4)	292.3 (3.93)	1412 (34.7)	235.3 (5.78)	<.001^e^	0.02^f^
Antiplatelets and anticoagulants	6940 (51.6)	694 (5.16)	3873 (52.2)	430.3 (5.8)	2115 (51.9)	352.5 (8.65)	.38^e^	—
Beta blockers	3311 (24.6)	331.1 (2.46)	569 (7.7)	63.2 (0.86)	52 (1.3)	8.7 (0.22)	<.001^e^	0.27^f^
Bronchodilators	1807 (13.4)	180.7 (1.34)	1117 (15)	124.1 (1.67)	813 (20)	135.5 (3.33)	<.001^e^	0.07^f^
Calcium channel blocker	5867 (43.6)	586.7 (4.36)	3240 (43.6)	360 (4.84)	1727 (42.4)	287.8 (7.07)	.44^e^	—
Diuretics	2412 (17.9)	241.2 (1.79)	1326 (17.9)	147.3 (1.99)	912 (22.4)	152 (3.73)	<.001^e^	0.04^f^
Inhaled corticosteroids	1013 (7.5)	101.3 (0.75)	540 (7.3)	60 (0.81)	359 (8.8)	59.8 (1.47)	.007^e^	0.02^f^
Rheumatoid drugs	1358 (10.1)	135.8 (1.01)	936 (12.6)	104 (1.4)	441 (10.8)	73.5 (1.8)	<.001^e^	0.04^f^
Statins	5901 (43.8)	590.1 (4.38)	3701 (49.9)	411.2 (5.54)	1993 (48.9)	332.2 (8.15)	<.001^e^	0.06^f^
Systemic corticosteroids	3665 (27.2)	366.5 (2.72)	2622 (35.3)	291.3 (3.92)	1491 (36.6)	248.5 (6.1)	<.001^e^	0.09^f^
Blood biomarkers, median (IQR)								
Albumin (g/L)	36.80 (7.90)	35.8 (1.3)	36.30 (7.50)	35.4 (0.3)	35.60 (8.20)	34.6 (0.3)	<.001^g^	0.004^h^
Neutrophil (×10^9^/L)	4.62 (3.57)	5.7 (0.5)	5.18 (3.69)	6.1 (0.2)	5.86 (4.47)	6.9 (0.08)	<.001^g^	0.02^h^
Bilirubin (µmol/L)	8.80 (7.20)	11.7 (0.7)	9 (7)	11.5 (0.6)	9 (7.20)	12.5 (0.5)	.16^g^	—
Lymphocyte (×10^9^/L)	0.99 (0.74)	1.4 (0.07)	0.90 (0.79)	1.5 (0.2)	1 (0.83)	1.5 (0.09)	<.001^g^	0.001^h^
Platelet (×10^9^/L)	201 (104.15)	215.9 (7.9)	195 (103)	209 (4.2)	206 (108)	222.8 (2.8)	<.001^g^	0.002^h^

a Episodes=13,549 and head count=11,517.

b Episodes=7424 and head count=6848.

c Episodes=4072 and head count=3760.

d Not available.

e Proportions test.

f Cramér V test.

g Kruskal-Wallis test.

h Eta squared test.

**Table 5.** Characteristics of patients infected with Omicron in Hong Kong from this repeated cross-sectional study (2022‐2024), stratified by ages 75‐84 years (total episodes, N=29,743).

Characteristics	Period 1^a^		Period 2^b^		Period 3^c^		*P* value	Effect size
	Observed	Adjusted	Observed	Adjusted	Observed	Adjusted		
Case fatality ratio	7.5	—^d^	7.3	—	7.5	—	—	—
≥2 hospital readmissions, n (%)	4845 (31)	484.5 (3.1)	1453 (16.1)	161.4 (1.79)	915 (18)	152.5 (3)	<.001^e^	0.57^e^
Sex, n (%)								
Male	9265 (59.3)	926.5 (5.93)	5280 (58.4)	586.7 (6.49)	2890 (58.7)	481.7 (9.78)	.01^f^	0.02^e^
Female	6357 (40.7)	635.7 (4.07)	3765 (41.6)	418.3 (4.62)	2096 (41.3)	349.3 (6.88)	.01^f^	0.02^e^
Comorbidities, n (%)								
Essential primary hypertension	958 (6.1)	95.8 (0.61)	625 (6.9)	69.4 (0.77)	320 (6.3)	53.3 (1.05)	.05^f^	—
Type 2 diabetes mellitus	884 (5.7)	88.4 (0.57)	573 (6.3)	63.7 (0.7)	269 (5.3)	44.8 (0.88)	<.001^f^	0.01^e^
Hyperlipidemia (unspecified)	372 (2.4)	37.2 (0.24)	293 (3.2)	32.6 (0.36)	156 (3.1)	26 (0.52)	<.001^f^	0.02^e^
Race, n (%)								
Chinese	15,178 (97.2)	1517.8 (9.72)	8752 (96.8)	972.4 (10.8)	4902 (96.6)	817 (16.1)	.06^f^	—
Non-Chinese	444 (2.8)	44.4 (0.28)	293 (3.2)	32.6 (0.36)	174 (3.4)	29 (0.57)	.06^f^	—
Length of hospital stay (days), median (IQR)	6 (8)	10.5 (0.4)	5 (6)	9.1 (1.1)	4 (6)	7.9 (2.2)	<.001^g^	0.01^h^
Charlson Comorbidity Index score, n (%)								
0	11,354 (72.7)	1135.4 (7.27)	6639 (73.5)	737.7 (8.17)	3558 (71.3)	593 (11.88)	<.001^f^	0.02^e^
1	2327 (14.9)	232.7 (1.49)	1401 (15.5)	155.7 (1.72)	815 (16.3)	135.8 (2.72)	.11^f^	—
2‐3	1560 (10)	156 (1.0)	774 (8.6)	86 (0.96)	482 (9.7)	80.3 (1.62)	.001^f^	0.02^e^
≥4	372 (2.4)	37.2 (0.24)	223 (2.5)	24.8 (0.28)	136 (2.7)	22.7 (0.45)	.49^f^	—
Frailty-related episodes, n (%)	1306 (8.4)	130.6 (0.84)	750 (8.3)	83.3 (0.92)	420 (8.3)	70 (1.38)	.97^f^	—
Social deprivation index, n (%)								
1 (least disadvantaged)	2187 (14)	218.7 (1.4)	1315 (14.6)	146.1 (1.62)	671 (13.3)	111.8 (2.22)	.09^f^	—
2 (slightly disadvantaged)	5247 (33.7)	524.7 (3.37)	3039 (33.6)	337.7 (3.73)	1831 (36.2)	305.2 (6.03)	.003^f^	0.02^e^
3 (moderately disadvantaged)	4560 (29.3)	456 (2.93)	2503 (27.7)	278.1 (3.08)	1337 (26.4)	222.8 (4.4)	<.001^f^	0.02^e^
4 (most disadvantaged)	3589 (23)	358.9 (2.3)	2177 (24.1)	241.9 (2.68)	1219 (24.1)	203.2 (4.02)	.09^f^	—
Drug administration, n (%)								
Angiotensin-converting enzyme inhibitors	6016 (38.5)	601.6 (3.85)	3593 (39.7)	399.2 (4.41)	2044 (40.3)	340.7 (6.72)	.04^f^	0.01^e^
Antidiabetics	5439 (34.8)	543.9 (3.48)	3268 (36.1)	363.1 (4.01)	1844 (36.3)	307.3 (6.05)	.04^f^	0.01^e^
Antiplatelets and anticoagulants	9859 (63.1)	985.9 (6.31)	5696 (63)	632.9 (7)	3171 (62.5)	528.5 (10.42)	.71^f^	—
Beta blockers	4319 (27.6)	431.9 (2.76)	735 (8.1)	81.7 (0.9)	85 (1.7)	14.2 (0.28)	<.001^f^	0.29^e^
Bronchodilators	2745 (17.6)	274.5 (1.76)	1891 (20.9)	210.1 (2.32)	1260 (24.8)	210 (4.13)	<.001^f^	0.07^e^
Calcium channel blocker	8185 (54.2)	818.5 (5.42)	4613 (51)	512.6 (5.67)	2589 (51)	431.5 (8.5)	.06^f^	—
Diuretics	3316 (21.2)	331.6 (2.12)	2011 (22.2)	223.4 (2.47)	1315 (25.9)	219.2 (4.32)	<.001^f^	0.04^e^
Inhaled corticosteroids	1296 (8.3)	129.6 (0.83)	813 (9)	90.3 (1)	503 (9.9)	83.8 (1.65)	.001^f^	0.02^e^
Rheumatoid drugs	1894 (12.1)	189.4 (1.21)	1234 (13.6)	137.1 (1.51)	649 (12.8)	108.2 (2.13)	.003^f^	0.02^e^
Statins	7839 (50.2)	783.9 (5.02)	5044 (55.8)	560.4 (6.2)	2876 (56.7)	479.3 (9.45)	<.001^f^	0.06^e^
Systemic corticosteroids	4881 (31.2)	488.1 (3.12)	3460 (38.3)	384.4 (4.26)	1907 (37.6)	317.8 (6.27)	<.001^f^	0.07^e^
Blood biomarkers, median (IQR)								
Albumin (g/L)	35.20 (8.02)	34.6 (1.1)	35 (7.90)	34.4 (0.4)	34.50 (8.50)	33.8 (0.4)	<.001^g^	0.002^h^
Neutrophil (×10^9^/L)	4.83 (3.70)	5.9 (0.5)	5.28 (3.77)	6.2 (0.2)	5.70 (4.24)	6.7 (0.2)	<.001^g^	0.01^h^
Bilirubin (µmol/L)	9 (7.10)	11.3 (0.5)	9.20 (7.20)	12.1 (0.4)	9 (6.80)	11.3 (0.3)	.03^g^	0.002^h^
Lymphocyte (×10^9^/L)	0.98 (0.72)	1.4 (0.1)	0.90 (0.77)	1.7 (0.3)	1(0.84)	1.7 (0.2)	<.001^g^	0.002^h^
Platelet (×10^9^/L)	194 (102)	210.9 (7.6)	189 (96)	203.7 (3.9)	200 (108)	217.1 (3.2)	<.001^g^	0.002^h^

a Episodes=15,622 and head count=12,935.

b Episodes=9045 and head count=8266.

c Episodes=5076 and head count=4572.

d Not available.

e Cramér V test.

f Proportions test.

g Kruskal-Wallis test.

h Eta squared test.

**Table 6.** Characteristics of patients infected with Omicron in Hong Kong from this repeated cross-sectional study (2022‐2024), stratified by ages older than 85 years (total episodes, N=39,107).

Characteristics	Period 1^a^		Period 2^b^		Period 3^c^		*P* value	Effect size
	Observed	Adjusted	Observed	Adjusted	Observed	Adjusted		
Case fatality ratio	11.6	—^d^	10.1	—	10.1	—	—	—
≥2 hospital readmissions, n (%)	7540 (38.2)	754 (3.82)	2626 (21.8)	291.8 (2.42)	1639 (22.3)	273.2 (3.72)	<.001^e^	0.48^f^
Sex, n (%)								
Male	8444 (42.8)	844.4 (4.28)	5152 (42.7)	572.4 (4.74)	3161 (43.1)	526.8 (7.18)	.89^e^	—
Female	11,273 (57.2)	1127.3 (5.72)	6901 (57.3)	766.8 (6.37)	4176 (56.9)	696 (9.48)	.89^e^	—
Comorbidities, n (%)								
Essential primary hypertension	1369 (6.9)	136.9 (0.69)	910 (7.5)	101.1 (0.83)	509 (6.9)	84.8 (1.15)	.10^e^	—
Type 2 diabetes mellitus	933 (4.7)	93.3 (0.47)	550 (4.6)	61.1 (0.51)	264 (3.6)	44 (0.6)	<.001^e^	0.02^f^
Hyperlipidemia (unspecified)	418 (2.1)	41.8 (0.21)	299 (2.5)	33.2 (0.28)	192 (2.6)	32 (0.43)	.02^e^	0.01^f^
Race, n (%)								
Chinese	19,356 (98.2)	1935.6 (9.82)	11,832 (98.2)	1314.7 (10.91)	7193 (98)	1198.8 (16.3)	.76^e^	—
Non-Chinese	361 (1.8)	36.1 (0.18)	221 (1.8)	24.6 (0.2)	144 (2)	24 (0.33)	.76^e^	—
Length of hospital stay (days), median (IQR)	7 (9)	10.1 (0.8)	5 (6)	8.7 (1.1)	5 (5)	7.6 (1.7)	<.001^g^	0.02^h^
Charlson Comorbidity Index score, n (%)								
0	14,820 (75.2)	1482 (7.52)	9364 (77.7)	1040.4 (8.63)	5576 (77.3)	929.3 (12.88)	<.001^e^	0.02^f^
1	2952 (15)	295.2 (1.5)	1696 (14.1)	188.4 (1.57)	1014 (14.1)	169 (2.35)	.02^e^	0.01^f^
2‐3	1617 (8.2)	161.7 (0.82)	810 (6.7)	90 (0.74)	529 (7.3)	88.2 (1.22)	<.001^e^	0.02^f^
≥4	310 (1.6)	31 (0.16)	178 (1.5)	19.8 (0.17)	98 (1.4)	16.3 (0.23)	.35^e^	—
Frailty-related episodes, n (%)	1903 (9.7)	190.3 (0.97)	1095 (9.1)	121.7 (1.01)	633 (8.6)	105.5 (1.43)	.02^e^	0.01^f^
Social deprivation index								
1 (least disadvantaged)	2715 (13.8)	271.5 (1.38)	1717 (14.3)	190.8 (1.59)	956 (13)	159.3 (2.17)	.06^e^	—
2 (slightly disadvantaged)	6294 (32)	629.4 (3.2)	3671 (30.5)	407.9 (3.39)	2327 (31.8)	387.8 (5.3)	.02^e^	0.01^f^
3 (moderately disadvantaged)	6016 (30.6)	601.6 (3.06)	3656 (30.4)	406.2 (3.38)	2184 (29.8)	364 (4.97)	.50^e^	—
4 (most disadvantaged)	4666 (23.7)	466.6 (2.37)	3001 (24.9)	333.4 (2.77)	1860 (25.4)	310 (4.23)	.004^e^	0.02^f^
Drug administration, n (%)								
Angiotensin-converting enzyme inhibitors	6981 (35.4)	698.1 (3.54)	4419 (36.7)	491 (4.08)	2656 (36.2)	442.7 (6.03)	.07^e^	—
Antidiabetics	4840 (24.5)	484 (2.45)	3140 (26.1)	348.9 (2.9)	1932 (26.3)	322 (4.38)	.001^e^	0.02^f^
Antiplatelets and anticoagulants	13,461 (68.3)	1346.1 (6.83)	8039 (66.7)	893.2 (7.41)	4851 (66.1)	808.5 (11.02)	<.001^e^	0.02^f^
Beta blockers	4823 (24.5)	482.3 (2.45)	945 (7.8)	105 (0.87)	104 (1.4)	17.3 (0.23)	<.001^e^	0.27^f^
Bronchodilators	3790 (19.2)	379 (1.92)	2698 (22.4)	299.8 (2.49)	1934 (26.4)	322.3 (4.4)	<.001^e^	0.09^f^
Calcium channel blocker	10,906 (55.3)	1090.6 (5.53)	6713 (55.7)	745.9 (6.19)	4024 (54.8)	670.7 (9.13)	.51^e^	—
Diuretics	4996 (25.3)	499.6 (2.53)	2968 (24.6)	329.8 (2.73)	2144 (29.2)	357.3 (4.87)	<.001^e^	0.04^f^
Inhaled corticosteroids	1442 (7.3)	144.2 (0.73)	918 (7.6)	102 (0.84)	615 (8.4)	102.5 (1.4)	.01^e^	0.01^f^
Rheumatoid drugs	2472 (12.5)	247.2 (1.25)	1596 (13.2)	177.3 (1.47)	972 (13.2)	162 (2.2)	.11^e^	—
Statins	8400 (42.6)	840 (4.26)	5783 (48)	642.6 (5.33)	3565 (48.6)	594.2 (8.1)	<.001^e^	0.06^f^
Systemic corticosteroids	6769 (34.3)	676.9 (3.43)	4951 (41.1)	550.1 (4.57)	2882 (39.3)	480.3 (6.55)	<.001^e^	0.06^f^
Blood biomarkers, median (IQR)								
Albumin (g/L)	33.30 (8.10)	32.8 (1.1)	33.70 (7.80)	33.1 (0.3)	32.90 (8.20)	32.3 (0.3)	<.001^g^	0.002^h^
Neutrophil (×10^9^/L)	5 (3.85)	6.1 (0.6)	5.44 (4)	6.5 (0.2)	5.92 (4.37)	7 (0.2)	<.001^g^	0.01^h^
Bilirubin (µmol/L)	9 (7.40)	11.3 (0.4)	9 (7)	11 (0.3)	9 (7.30)	11.1 (0.3)	.01^g^	0.0002^h^
Lymphocyte (×10^9^/L)	1 (0.75)	1.4 (0.05)	0.99 (0.80)	1.6 (0.04)	1.03 (0.87)	1.7 (0.1)	<.001^g^	0.001^h^
Platelet (×10^9^/L)	193 (103)	210.1 (9)	187 (99)	204.6 (4.5)	197 (104)	213.6 (2.5)	<.001^g^	0.001^h^

a Episodes=19,717 and head count=15,529.

b Episodes=12,053 and head count=10,639.

c Episodes=7337 and head count=6456.

d Not available.

e Proportions test.

f Cramér V test.

g Kruskal-Wallis test.

h Eta squared test.

### Demographic Profile of Hospitalized Patients Across Periods

Tables 2-6 and Table S1 in Multimedia Appendix 1 demonstrated demographic shifts across periods 1 to 3 by age subgroups. First, the CFR increased in each period as the age groups got older (Figure S1 in Multimedia Appendix 1) and lowered in patients older than 85 years (1.5% difference, period 1: 11.6%, period 3: 10.1%, *P*<.001, effect size: 0.02). Second, the monthly episodic rate of males and females hospitalized for COVID-19 infection decreased across periods for ages 0‐84. Females were predominantly accounted for infections in patients 18‐64, aged older than 85, whereas more males were infected in age groups 0‐17, 64‐74, and 75‐84 (Figure S2 in Multimedia Appendix 1).

Third, the proportion of individuals of the Chinese race dominated across all age groups, becoming more prominent as age increased (Figure S4 in Multimedia Appendix 1). Only those aged 0‐17 reported decreased proportions of Chinese race (8.5% difference, period 1: 77.2%, period 3: 68.7%, *P*<.001, effect size: 0.07). Also reflected in the adjusted measurements (410.1 episodes per month decrease, period 1: 648.3 episodes per month, period 2: 238.2 episodes per month).

Fourth, the Charlson Comorbidity Index scores in most age groups showed a predominant proportion of infected individuals with scores of 0 (more than 70% per period; Figure S5 in Multimedia Appendix 1), with notable decrease across the study period in age groups 0‐17 (0.5% difference, period 1: 98.1%, period 3: 97.6%, *P*=.003, effect size: 0.03; 486.4 episodes per month decrease, period 1: 823.6 episodes per month, period 3: 337.2 episodes per month), 18‐64 (2% difference, period 1: 78.5%, period 3: 76.5%, *P*<.001, effect size: 0.03; 653.7 episodes per month decrease, period 1: 1195 episodes per month, period 3: 541.3 episodes per month), 65‐74 (1.3% difference, period 1: 72.5%, period 3: 71.2%, *P*=.04, effect size: 0.01; 499.8 episodes per month decrease, period 1: 974 episodes per month, period 3: 474.2 episodes per month), 75‐84 (1.4% difference, period 1: 72.7%, period 3: 71.3%, *P*<.001, effect size: 0.02; 332 episodes per month decrease, period 1: 484.5 episodes per month, period 3: 152.5 episodes per month). In contrast, infected patients older than 85 showed an increase (2.1% difference, period 1: 75.2%, period 3: 77.3%, *P*<.001, effect size: 0.02), but the adjusted measurements showed otherwise (552.7 episodes per month decrease, period 1: 1482 episodes per month, period 3: 929.3 episodes per month). Although the proportion of infected patients with comorbid essential primary hypertension, type 2 diabetes mellitus, and hyperlipidemia (unspecified) remained below 10% and decreased in rate across periods and age groups (Figure S3 in Multimedia Appendix 1). Additionally, less than 10% of episodes per period and age group were related to frailty, although this proportion increased with age (Figure S8 in Multimedia Appendix 1). The rates decreased across periods for each age group.

Fifth, the distribution of the social deprivation index of infected individuals centered around slightly disadvantaged populations in Hong Kong (Figure S6 in Multimedia Appendix 1). This demographic showed higher proportions across periods among age groups 75‐84 (2.5% difference, period 1: 33.7%, period 3: 36.2%, *P*=.003, effect size: 0.02) and lower proportions in those older than 85 years (0.2% difference, period 1: 32%, period 3: 31.8%, *P*=.02, effect size: 0.01). Although both age groups decreased in rate across period (age group 75‐84: 219.5 episodes per month decrease, period 1: 524.7 episodes per month, period 3: 305.2 episodes per month; age group older than 85 years: 241.6 episodes per month decrease, period 1: 629.4 episodes per month, period 3: 387.8 episodes per month).

### Clinical Changes in Hospitalized Patients Across Periods

The findings from Tables 2-6 and Table S1 in Multimedia Appendix 1 also showed changes in the clinical management of hospitalized patients infected with Omicron across the study period when stratified by age groups. First, the length of hospital stay decreased across periods for age groups 18‐64 (1 d decrease, period 1: 4 d, period 3: 3 d, *P*<.001, effect size: 0.007; 4.1 d per month decrease, period 1: 11 d per month, period 3: 6.9 d per month), 65‐74 (2 d decrease, period 1: 6 d, period 3: 4 d, *P*<.001, effect size: 0.01; 2.6 d per month decrease, period 1: 10.4 d per month, period 3: 7.8 d per month), 75‐84 (2 d decrease, period 1: 6 d, period 3: 4 d, *P*<.001, effect size: 0.01; 2.6 d per month decrease, period 1: 10.5 d per month, period 3: 7.9 d per month), and older than 85 (2 days decrease, period 1: 7 d, period 3: 5 d, *P*<.001, effect size: 0.02; 2.5 d per month decrease, period 1: 10.1 d per month, period 3: 7.6 d per month). Additionally, a longer length of stay was observed as the demographic aged (Figure S9 in Multimedia Appendix 1).

Second, calcium channel blockers, statins, antiplatelets, and anticoagulants were the most frequently administered to patients aged 18 and older (Figure S7 in Multimedia Appendix 1), but the rate of administration reduced over time. Moreover, patients 0‐17 years received 4.2% more bronchodilators over the course of the study period (period 1: 2.9%, period 3: 7.1%, *P*<.001, effect size: 0.08) and slightly increased rate of administration (0.4 episodes per month, period 1: 24.7 episodes per month decrease, period 3: 24.3 episodes per month); 5.5% more bronchodilators in 18‐64 years (period 1: 6.7%, period 3: 12.2%, *P*<.001, effect size: 0.07) and decreased rate of administration (13.4 episodes per month decrease, period 1: 101.7 episodes per month, period 3: 88.3 episodes per month); 6.6% more bronchodilators in 65‐74 years (period 1: 13.4%, period 3: 20%, *P*<.001, effect size: 0.07) and decreasing rate of administration across periods (45.2 episodes per month decrease, period 1: 180.7 episodes per month, period 3: 135.5 episodes per month); 7.2% more bronchodilators in 75‐84 years (period 1: 17.6%, period 3: 24.8%, *P*<.001, effect size: 0.07) and decreasing rate of administration (64.5 episodes per month decrease, period 1: 274.5 episodes per month, period 3: 210 episodes per month); and 7.2% more bronchodilators in older than 85 (period 1:19.2%, period 3: 26.4%, *P*<.001, effect size: 0.09) and decreasing rate of administration (56.7 episodes per month decrease, period 1: 379 episodes per month, period 3: 322.3 episodes per month). Meanwhile, systemic corticosteroids were administered the most frequently in the 18‐64 age group (6.8% difference, period 1: 18.8%, period 3: 25.6%, *P*<.001, effect size: 0.09) with decreasing rate of administration (101.2 episodes per month decrease, period 1: 286.7 episodes per month, period 3: 185.5 episodes per month); and the 65‐74 age group (9.4% difference, period 1: 27.2%, period 3: 36.6%, *P*<.001, effect size: 0.09) with decreasing rate of administration (118 episodes per month, period 1: 366.5 episodes per month, period 3: 248.5 episodes per month). The highest effect size was observed with the decline in the proportion of patients administered beta blockers over time (effect size in aged 0‐17: 0.52, 64‐74: 0.27, 75‐84: 0.29, older than 85: 0.27).

Third, all the blood biomarkers exhibited minimal changes across periods in each age stratification (Figure S10 in Multimedia Appendix 1). Median albumin levels lowered, and median neutrophil levels elevated as age groups got older, especially in patients older than 85 years (albumin: 32.9 to 38.82g/L and 37.3 to 36.7g/L per month; neutrophil: 4.48 to 5.92×10^9^/L and 5.6 to 6.4×10^9^/L per month). Meanwhile, median bilirubin, lymphocyte, and platelet levels fluctuated across age groups.

## Discussion

### Principal Findings

To our knowledge, this is among the first retrospective, repeated cross-sectional studies to review changes in demographic and clinical characteristics of all patients infected with Omicron admitted to public hospitals in Hong Kong since the Omicron outbreak, including postpandemic periods. Additionally, this study expanded upon existing literature by providing insight into all age groups and incorporating social deprivation index scores.

### Trends in Hospitalizations and Case Fatality Rate

Although the epidemic curves demonstrated a decrease in the number of hospitalized Omicron cases, this study observed an increase in the CFR with advancing age, which was consistent with reports from other local and international studies [34,35]. In contrast, the CFR started to decline among patients aged older than 85 years, a trend not observed in previous studies. This finding suggested there were age-related disparities among Omicron infections. Furthermore, the rate of hospital readmission decreased across periods for all ages, which contradicted the findings from a study in the UK [36]. They suggested that recent vaccination reduced the risk of reinfection, and subsequent reinfections demonstrated lower severity [36], but further research is needed to ascertain this in our population group.

### Comorbidity Differences

The shift in Omicron infections to patients with more than 1 CCI over time aligned with local and international studies conducted in a postpandemic setting. They further attributed this shift to prolonged viral shedding from SARS-CoV-2 rebound [37-40], but subsequent research is needed to ascertain this hypothesis in our study population. Namely, our findings indicated that the proportion of patients with hypertension and hyperlipidemia comorbidities was low across periods, even though past literature suggested that existing low-grade chronic systemic inflammatory diseases can complicate clinical management of infected patients and increase the risk of poorer outcomes [41].

### Gender Differences

Our results also indicated a predominance of male patients in most age groups (0‐17 y, 64‐74 y, and 75‐84 y) throughout the study period, an observation that was well established during the pandemic [42]. Several hypotheses were proposed: one was the lower uptake of the second dose of COVID-19 vaccines among males [43], while another hypothesized a link to cardiovascular factors [43].

### Socioeconomic Demographic Shift

Most infections in our study population occurred among individuals from slightly disadvantaged backgrounds, even though the rate decreased across periods. Few studies explored the impact of the Omicron outbreak on patients from varied socioeconomic statuses. A study conducted during the early phase of COVID-19 in Hong Kong reported an association between socioeconomic disadvantage and a broader spread of infections [44], and was particularly related to essential activities such as living and working. International studies similarly found an association with lower socioeconomic status, hypothesizing that vaccine coverage might be an underlying reason [45,46].

### Changes in In-Hospital Drug Administrations

Systemic corticosteroids became an increasingly common and effective treatment for COVID-19, as demonstrated by the rising proportion and reduced rate of administration among patients aged 18 years to 74 years and across periods in our findings, as well as in other studies [47,48]. One study suggested that while such treatments have anti-inflammatory effects, this could induce immunosuppression, potentially delaying viral clearance and increasing the risk of poorer outcomes [49]. This led to differing clinical management recommendations, with the WHO advising against corticosteroid treatments in severe cases [50], while local and Chinese studies recommended low-dose, short-course treatments for severe cases [51,52].

Also, cardiovascular treatments, including calcium channel blockers, statins, antiplatelets, and anticoagulants, were the most frequently administered, with a reduced rate of administration among adults and older individuals in this study. Global studies established a relationship between COVID-19 and cardiovascular complications [53,54]. In particular, studies from China reported a high prevalence of myocardial injury among infected patients with poor prognosis [55]. To mitigate cardiovascular complications, subsequent studies demonstrated improved prognosis in patients with COVID-19 administered with calcium channel blockers [56,57], statins [58], anticoagulants [59], and antiplatelet agents [60]. Therefore, our findings may reflect the common clinical treatment for Omicron cases.

Additionally, we observed an increased use of bronchodilators and a decreasing rate of administration over time. This might have reflected evolving clinical management strategies rather than increased severity, as by 2022, over 90% of the Hong Kong population was vaccinated, and the CFR in our study remained largely static across all age groups [61]. However, further research was required to clarify these observations.

### Changes in Biomarker Indicators

Recent studies aligned with our findings and further demonstrated an association with COVID-19-related mortality. For example, one study found that elevated neutrophil levels were associated with increased COVID-19 mortality [62], while another study from China showed that hypoalbuminemia (albumin <35g/L) increased the odds of mortality [63]. Therefore, further research can help better understand the clinical management of infected patients, particularly in those aged over 85 years.

### Age Disparities in Omicron Cases

Older age saw a higher case-mortality ratio, likely from the higher CCI scores and frailty also observed in other studies [21,64], making this demographic more susceptible to infection. This was possibly exacerbated by the excess mortality among the older individuals during the Omicron outbreak in Hong Kong [65], the largest local outbreak to date. Such age disparities were reported to persist in the postpandemic setting [65], although differences between age groups appeared to have narrowed. However, the EuCARE-HOSPITALISED international study observed opposite trends [66]. This may be attributed to Hong Kong’s low vaccine uptake during the Omicron outbreak, but further research was needed to confirm this hypothesis.

### Limitations

Although this study collected a large sample size from all the public hospitals in Hong Kong, several limitations remained. First, our sample is not representative of all cases of COVID-19 infection in Hong Kong as it did not include unreported and asymptomatic cases, data from private hospitals, or non-Hong Kong residents. Second, our SDI score was based on calculations from 2008. Such definitions may be outdated and differ from the current situation in Hong Kong. The SDI distribution may also be skewed towards more disadvantaged populations with the exclusion of private hospital data. So, the respective findings from Tables 2-6 should be interpreted with caution. Third, the list of frailty-related diagnoses was defined by clinicians from an acute hospital trust in England, which may not be representative of frail patients in Hong Kong. Therefore, our findings should be interpreted with caution. Fourth, the patient’s vaccination status was not available, so we cannot understand the impact of vaccination on incidence.

### Conclusions

Our study provides an updated descriptive overview of postpandemic Omicron hospitalizations in Hong Kong. The findings highlight the need for age-specific interventions, particularly among older individuals. Further research is essential to understand the effectiveness of the vaccine booster dose in a postpandemic setting. All to improve pandemic preparedness and to develop more effective public health strategies in the ongoing fight against COVID-19.

## Supplementary material

10.2196/75635 Multimedia Appendix 1 Drugs and post hoc analysis characterizing patients infected with Omicron.

## Abbreviations

CCI: Charlson Comorbidity Index
CFR: case fatality ratio
*ICD-10*: *International Statistical Classification of Diseases and Related Health Problems 10th Revision*
SDI: Social Deprivation Index
WHO: World Health Organization
