# Supplementary material for: Postpandemic Change in Demographic and Clinical Features of Patients With Omicron Who Were Hospitalized: Territory-Wide Retrospective Repeated Cross-Sectional Study in Hong Kong
Source: JMIR Public Health Surveill. 2026 Feb 9;12:e75635. doi: 10.2196/75635 (PMC12885189; doi:10.2196/75635)
Supplement: Multimedia Appendix 1 [file publichealth-v12-e75635-s001.docx]

**Table S1.** Post-hoc analysis of characteristics of Omicron infected patients hospitalized in Hong Kong between 2022 and 2024 in this repeated cross-sectional study, stratified by (a) 0-17 years old, (b) 18-64 years old, (c) aged 65-74 years old, (d) aged 75-84 years old, (e) aged older than 85 years old. P-values adjusted with Bonferroni correction.

(a)

|  | | **Period 1 & 2** | **Period 2 & 3** | **Period 1 & 3** |
| --- | --- | --- | --- | --- |
| **Sex** | | 0.02 | 1.00 | 0.57 |
| **Race** | | <0.001 | <0.001 | <0.001 |
| **Length of Hospital Stay (days)** | | <0.001 | 1.00 | <0.001 |
| **Charlson Comorbidity Index Score** | | | | |
|  | 0 | 0.009 | 1.00 | 0.06 |
|  | 2-3 | 0.05 | 1.00 | 0.82 |
| **Social Deprivation Index** | | | | |
|  | 1 (Least disadvantaged) | 0.06 | 1.00 | 0.50 |
|  | 3 (Moderately disadvantaged) | 0.004 | 1.00 | 0.03 |
|  | 4 (Most disadvantaged) | 0.10 | 1.00 | 0.03 |
| **Drug Administration** | |  |  |  |
|  | Antiplatelets and Anticoagulants | 0.04 | 1.00 | 0.11 |
|  | Beta Blockers | 0.17 | 1.00 | 0.09 |
|  | Bronchodilators | <0.001 | 0.03 | <0.001 |
|  | Calcium Channel Blockers | 0.04 | 0.09 | 1.00 |
|  | Diuretics | 0.03 | 1.00 | 0.24 |
|  | Inhaled Corticosteroids | 0.04 | 0.02 | <0.001 |
| **Blood Biomarkers** | |  |  |  |
|  | Albumin (g/L) | <0.001 | 0.81 | <0.001 |
|  | Neutrophil (x10^9^/L) | 0.009 | 1.00 | 0.004 |
|  | Lymphocyte (x10^9^/L) | <0.001 | <0.001 | <0.001 |
|  | Platelet (x10^9^/L) | <0.001 | <0.001 | <0.001 |

*a Pairwise Proportions Test*

*b Pairwise Fishers Exact Test
c Dunn’s Test*

(b)

|  |  | **Period 1 & 2** | **Period 2 & 3** | **Period 1 & 3** |
| --- | --- | --- | --- | --- |
| **Sex** | | <0.001 | 1.00 | 0.07 |
| **Essential Primary Hypertension** | | 0.09 | 0.89 | 0.01 |
| **Length of Hospital Stay (days)** | | <0.001 | <0.001 | <0.001 |
| **Charlson Comorbidity Index Score** | | | | |
|  | 0 | 1.00 | <0.001 | <0.001 |
|  | 1 | 0.01 | 0.70 | <0.001 |
| **Drug Administration** | |  |  |  |
|  | ACE Inhibitors | <0.001 | 1.00 | 0.003 |
|  | Antidiabetics | <0.001 | 1.00 | 0.03 |
|  | Antiplatelets and Anticoagulants | 0.12 | 1.00 | 0.43 |
|  | Beta Blockers | <0.001 | <0.001 | <0.001 |
|  | Bronchodilators | <0.001 | <0.001 | <0.001 |
|  | Calcium Channel Blockers | 0.007 | 0.94 | 0.001 |
|  | Diuretics | 0.07 | 0.04 | <0.001 |
|  | Inhaled Corticosteroids | 0.40 | 0.18 | 0.003 |
|  | Rheumatoid Drugs | <0.001 | 1.00 | <0.001 |
|  | Statins | <0.001 | 1.00 | <0.001 |
|  | Systemic Corticosteroids | <0.001 | 0.19 | <0.001 |
| **Blood Biomarkers** | |  |  |  |
|  | Albumin (g/L) | <0.001 | 0.20 | <0.001 |
|  | Neutrophil (x10^9^/L) | <0.001 | <0.001 | <0.001 |
|  | Bilirubin (umol/L) | <0.001 | 1.00 | <0.001 |
|  | Lymphocyte (x10^9^/L) | <0.001 | <0.001 | 0.05 |
|  | Platelet (x10^9^/L) | 0.97 | <0.001 | <0.001 |

*a Pairwise Proportions Test
b Dunn’s Test*

(c)

|  |  | **Period 1 & 2** | **Period 2 & 3** | **Period 1 & 3** |
| --- | --- | --- | --- | --- |
| **Sex** | | <0.001 | 0.05 | 1.00 |
| **Comorbidities** | |  |  |  |
|  | Essential Primary Hypertension | 0.002 | 1.00 | 0.26 |
|  | Type 2 Diabetes Mellitus | <0.001 | 0.002 | 1.00 |
|  | Hyperlipidemia (Unspecified) | 0.004 | 1.00 | 0.35 |
| **Race** | | 0.008 | 1.00 | 0.39 |
| **Length of Hospital Stay (days)** | | <0.001 | 1.00 | <0.001 |
| **Charlson Comorbidity Index Score** | | | | |
|  | 0 | 0.90 | 0.42 | 0.04 |
|  | 1 | <0.001 | 0.57 | 0.37 |
| **Frailty Related Episodes** | | <0.001 | 0.03 | 1.00 |
| **Drug Administration** | |  |  |  |
|  | Antidiabetics | <0.001 | 1.00 | 0.10 |
|  | Beta Blockers | <0.001 | <0.001 | <0.001 |
|  | Bronchodilators | 0.002 | <0.001 | <0.001 |
|  | Diuretics | 1.00 | <0.001 | <0.001 |
|  | Inhaled Corticosteroids | 1.00 | 0.11 | 0.02 |
|  | Rheumatoid Drugs | <0.001 | 0.02 | 0.43 |
|  | Statins | <0.001 | 1.00 | <0.001 |
|  | Systemic Corticosteroids | <0.001 | 0.51 | <0.001 |
| **Blood Biomarkers** | |  |  |  |
|  | Albumin (g/L) | <0.001 | <0.001 | <0.001 |
|  | Neutrophil (x10^9^/L) | <0.001 | <0.001 | <0.001 |
|  | Lymphocyte (x10^9^/L) | <0.001 | <0.001 | 1.00 |
|  | Platelet (x10^9^/L) | <0.001 | <0.001 | <0.001 |

*a Pairwise Proportions Test
b Dunn’s Test*

(d)

|  |  | **Period 1 & 2** | **Period 2 & 3** | **Period 1 & 3** |
| --- | --- | --- | --- | --- |
| **Sex** | | 0.47 | 0.30 | 0.009 |
| **Comorbidities** | |  |  |  |
|  | Type 2 Diabetes Mellitus | 0.008 | <0.001 | <0.001 |
|  | Hyperlipidemia (Unspecified) | <0.001 | 1.00 | 0.02 |
| **Length of Hospital Stay (days)** | | <0.001 | <0.001 | <0.001 |
| **Charlson Comorbidity Index Score** | | | | |
|  | 0 | 0.68 | <0.001 | 0.001 |
|  | 2-3 | <0.001 | 0.19 | 0.97 |
| **Social Deprivation Index** | | | | |
|  | 2 (Slightly disadvantaged) | 1.00 | 0.01 | 0.004 |
|  | 3 (Moderately disadvantaged) | 0.03 | 0.27 | <0.001 |
| **Drug Administration** | |  |  |  |
|  | ACE Inhibitors | 0.18 | 1.00 | 0.08 |
|  | Antidiabetics | 0.12 | 1.00 | 0.16 |
|  | Beta Blockers | <0.001 | <0.001 | <0.001 |
|  | Bronchodilators | <0.001 | <0.001 | <0.001 |
|  | Diuretics | 0.20 | <0.001 | <0.001 |
|  | Inhaled Corticosteroids | 0.19 | 0.23 | 0.001 |
|  | Rheumatoid Drugs | 0.002 | 0.47 | 0.66 |
|  | Statins | <0.001 | 0.94 | <0.001 |
|  | Systemic Corticosteroids | <0.001 | 1.00 | <0.001 |
| **Blood Biomarkers** | |  |  |  |
|  | Albumin (g/L) | 0.03 | <0.001 | <0.001 |
|  | Neutrophil (x10^9^/L) | <0.001 | <0.001 | <0.001 |
|  | Bilirubin (umol/L) | 0.10 | 0.04 | 1.00 |
|  | Lymphocyte (x10^9^/L) | <0.001 | <0.001 | 0.009 |
|  | Platelet (x10^9^/L) | <0.001 | <0.001 | <0.001 |

*a Pairwise Proportions Test
b Dunn’s Test*

(e)

|  |  | **Period 1 & 2** | **Period 2 & 3** | **Period 1 & 3** |
| --- | --- | --- | --- | --- |
| **Comorbidities** | | | | |
|  | Type 2 Diabetes Mellitus | 1.00 | 0.004 | <0.001 |
|  | Hyperlipidemia (Unspecified) | 0.12 | 1.00 | 0.05 |
| **Length of Hospital Stay (days)** | | <0.001 | <0.001 | <0.001 |
| **Charlson Comorbidity Index Score** | | | | |
|  | 0 | <0.001 | 0.02 | 0.48 |
|  | 1 | 0.09 | 2.00 | 0.06 |
|  | 2-3 | <0.001 | 0.61 | 0.02 |
| **Frailty Related Episodes** | | 0.29 | 0.87 | 0.03 |
| **Social Deprivation Index** | | | | |
|  | 2 (Slightly disadvantaged) | 0.02 | 0.20 | 1.00 |
|  | 4 (Most disadvantaged) | 0.04 | 1.00 | 0.01 |
| **Drug Administration** | |  |  |  |
|  | Antidiabetics | 0.009 | 1.00 | 0.008 |
|  | Antiplatelets and Anticoagulants | 0.01 | 1.00 | 0.002 |
|  | Beta Blockers | <0.001 | <0.001 | <0.001 |
|  | Bronchodilators | <0.001 | <0.001 | <0.001 |
|  | Diuretics | 0.47 | <0.001 | <0.001 |
|  | Inhaled Corticosteroids | 0.99 | 0.18 | 0.01 |
|  | Statins | <0.001 | 1.00 | <0.001 |
|  | Systemic Corticosteroids | <0.001 | 0.04 | <0.001 |
| **Blood Biomarkers** | |  |  |  |
|  | Albumin (g/L) | 0.004 | <0.001 | <0.001 |
|  | Neutrophil (x10^9^/L) | <0.001 | <0.001 | <0.001 |
|  | Bilirubin (umol/L) | 0.06 | 1.00 | 0.03 |
|  | Lymphocyte (x10^9^/L) | 1.00 | <0.001 | <0.001 |
|  | Platelet (x10^9^/L) | <0.001 | <0.001 | 0.004 |

*a Pairwise Proportions Test
b Dunn’s Test*

**Table S2.** Categorization of drugs administered to Omicron-infected patients admitted in-hospital in this repeated cross-sectional study (2022-2024).

| **Categories of Drugs** | **Drug names** |
| --- | --- |
| Rheumatoid Drugs | Allopurinol, Colchicine, Hydroxychloroquine, Sulfasalazine, Febuxostat, Rasburicase, Leflunomide, Baricitinib, Certolizumab, Tofacitinib, Etanercept, Tocilizumab, Adalimumab, Probenecid, Ixekizumab, Secukinumab, Ustekinumab, Belimumab, Golimumab |
| Systemic Corticosteroids | Prednisolone, Hydrocortisone, Dexamethasone, Fludrocortisone, Triamcinolone, Methylprednisolone |

**Figure S1.** Case fatality ratio of Omicron-infected patients admitted in-hospital in this repeated cross-sectional study (2022-2024), stratified by age group.

**Figure S2.** Proportion of sex of Omicron-infected patients admitted in-hospital in this repeated cross-sectional study (2022-2024), stratified by age group.

1. 0-17 Years Old
2. 18-64 Years Old
3. 65-74 years old
4. 75-84 years old

**Figure S3.** Percentage of comorbidities of Omicron-infected patients admitted in-hospital in this repeated cross-sectional study (2022-2024), stratified by age sub-groups and yielding significant difference across periods.

1. Essential Primary Hypertension
2. Type 2 Diabetes Mellitus
3. Hyperlipidemia (Unspecified)

**Figure S4.** Proportion of race of Omicron-infected patients admitted in-hospital in this repeated cross-sectional study (2022-2024), stratified by age groups and yielded significant change across periods.

1. 0-17 Years Old
2. 65-74 years old

**Figure S5.** Proportion of CCI of Omicron-infected patients admitted in-hospital in this repeated cross-sectional study (2022-2024), stratified by age group.

1. 0-17 Years Old
2. 18-64 years old
3. 65-74 years old
4. 75-84 years old
5. Older than 85 years old

**Figure S6.** Proportion of SDI of Omicron-infected patients admitted in-hospital in this repeated cross-sectional study (2022-2024), stratified by age group.

1. 0-17 Years Old
2. 18-64 years old
3. 65-74 years old
4. 75-84 years old
5. Older than 85 years old

**Figure S7.** Proportion of drugs administered of Omicron-infected patients admitted in-hospital in this repeated cross-sectional study (2022-2024), stratified by age group.

1. ACE Inhibitors
2. Antidiabetics
3. Antiplatelets and Anticoagulants
4. Beta Blockers
5. Bronchodilators
6. Calcium Channel Blockers
7. Diuretics
8. Inhaled Corticosteroids
9. Rheumatoid Drugs
10. Statins
11. Systemic Corticosteroids

**Figure S8.** Percentage of frailty related episodes of Omicron-infected patients admitted in-hospital in this repeated cross-sectional study (2022-2024), stratified by age group.

**Figure S9.** Change in length of stay of Omicron-infected patients admitted in-hospital in this repeated cross-sectional study (2022-2024), stratified by age group.

**Figure S10.** Change in blood biomarkers of Omicron-infected patients admitted in-hospital in this repeated cross-sectional study (2022-2024), stratified by age group.

1. Albumin
2. Neutrophil
3. Bilirubin
4. Lymphocyte

1. Platelet
